# Assessing glycemic variability in critically ill patients: A prospective cohort study comparing insulin infusion therapy with insulin sliding scale

**DOI:** 10.1038/s41598-024-57403-5

**Published:** 2024-05-02

**Authors:** Alaa Almagthali, Samiah Alsohimi, Arwa Alkhalaf, Khalid Al Sulaiman, Ohoud Aljuhani

**Affiliations:** 1 Pharmaceutical Care Department, King Abdulaziz University Hospital, Jeddah, Saudi Arabia; 2 Pharmaceutical Care Department, King Fahad Armed Forces hospital, Jeddah, Saudi Arabia; 3 Measurement and Psychometrics, Psychology Department, Faculty of Graduate Educational Studies, King Abdulaziz University, Jeddah, Saudi Arabia; 4 Pharmaceutical Care Department, King Abdulaziz Medical City, Riyadh, Saudi Arabia; 5 College of Pharmacy, King Saud Bin Abdulaziz University for Health Sciences, Riyadh, Saudi Arabia; 6 King Abdullah International Medical Research Center-King Saud Bin Abdulaziz University for Health Sciences, Ministry of National Guard-Health Affairs, Riyadh, Saudi Arabia; 7 Saudi Critical Care Pharmacy Research (SCAPE) Platform, Riyadh, Saudi Arabia; 8 Saudi Society for Multidisciplinary Research Development and Education (SCAPE Society), Riyadh, Saudi Arabia; 9 Department of Pharmacy Practice, Faculty of Pharmacy, King Abdulaziz University, Jeddah, Saudi Arabia

**Keywords:** Endocrine system and metabolic diseases, Outcomes research

## Abstract

Glycemic variability (GV) has been associated with an increased mortality rate among critically ill patients. The clinical outcomes of having less GV even with slight hyperglycemia are better than those having tight glycemic control but higher GV. Insulin infusion remains the preferred method to control stress hyperglycemia in critically ill patients. However, its impacts on GV and clinical outcomes in critically ill patients still need further investigation. This study intended to evaluate the impact of insulin infusion therapy (IIT) compared to the insulin sliding scale (ISS) on the extent of GV and explore its impact on the clinical outcomes for critically ill patients. A prospective, single-center observational cohort study was conducted at a tertiary academic hospital in Saudi Arabia between March 2021 and November 2021. The study included adult patients admitted to ICUs who received insulin for stress hyperglycemia management. Patients were categorized into two groups based on the regimen of insulin therapy during ICU stay (IIT versus ISS). The primary outcome was the GV between the two groups. Secondary outcomes were ICU mortality, the incidence of hypoglycemia, and ICU length of stay (LOS). A total of 381 patients were screened; out of them, eighty patients met the eligibility criteria. The distribution of patients having diabetes and a history of insulin use was similar between the two groups. The GV was lower in the IIT group compared to the ISS group using CONGA (− 0.65, 95% CI [− 1.16, − 0.14], *p*-value = 0.01). Compared with ISS, patients who received IIT had a lower incidence of hypoglycemia that required correction (6.8% vs 2.77%; *p*-value = 0.38). In contrast, there were no significant differences in ICU LOS and ICU mortality between the two groups. Our study demonstrated that the IIT is associated with decreased GV significantly in critically ill patients without increasing the incidence of severe hypoglycemia. There is no survival benefit with the use of the IIT. Further studies with larger sample size are required to confirm our findings and elaborate on IIT's potential effect in reducing ICU complications in critically ill patients.

## Introduction

Alteration of blood glucose (BG) homeostasis during critical illness is common^1^. Critically ill patients develop stress‐induced hyperglycemia (SIH) even without a history of diabetes. Common pathophysiological features of SIH include changes in hepatic glucose production, increased lipolysis, heightened insulin receptor sensitivity, alterations in hormone secretion, and activation of cytokines^2,3^. Moreover, SIH and hypoglycemia are associated with an increased morbidity and mortality rate^4–6^. Furthermore, some intensive care unit (ICU) management strategies increase the risk of developing hyperglycemia, including highly concentrated glucose intravenous fluids, catecholamine infusion, renal replacement therapy, and several medications such as; glucocorticoids^7^.

Glycemic control in ICU patients is recommended as the standard of care in critically ill patients^8^. In 2009, the NICE-Sugar (Normoglycaemia in Intensive Care Evaluation—Survival Using Glucose Algorithm Regulation) trial found that tight glycemic control was associated with significantly increased mortality in ICU patients. Following the NICE Sugar publication, the appropriate and safe goals for blood glucose for the ICU setting were determined to be 140 to 180 mg/dL (7.8–10 mmol/L). The Society of Critical Care Medicine (SCCM) for the management of hyperglycemia in critically ill patients recommends initiating intervention if the BG reading is 150 mg/dL (≥ 8.3 mmol/L) or more. Furthermore, conventional BG targets lower than 180 mg/dL (10 mmol/L) resulted in lower mortality compared to tight glycemic control. In addition, they recommend avoiding hypoglycemia less than 70 mg/dL (< 3.9 mmol/L) and glycemic variability (GV)^9^.

The literature supports the use of insulin infusion over the subcutaneous route of insulin administration for several clinical indications, including diabetic ketoacidosis (DKA), critical care illness accompanied by hemodynamic instability, and postcardiac surgical procedures^10–14^. Several factors may influence the subcutaneous blood flow that ultimately may impact the insulin absorption including the site of injection, temperature, obesity, body position, blood pressure, and use of vasodilating/vasoconstricting medications^15^. On the contrary, IIT is rarely impacted by these factors and rapidly achieves and maintains the target of the blood glucose^14^.

Glucose variability is an independent risk factor that increases infection rate, longer hospital, and ICU stays, as well as increased mortality^16^. It has been shown that high glucose variability is associated with ICU mortality by increased oxidative stress, mitochondrial damage, neuronal damage, and coagulation activity^17,18^. Similarly, hypoglycemia is related to mortality and prolonged hospital length of stay, reduced patient psychological well-being and quality of life, and an increased risk of cardiovascular events in the ICU and hospitalized patients with septic shock^19–22^. With this insight in mind, it is advisable to investigate strategies and treatment protocols aimed at mitigating glycemic variability in ICU patients.

GV describes the extent of BG oscillations, in both magnitude and frequency including episodes of hypoglycemia and hyperglycemia^23,24^. Eslami et al. noted that more than ten different measures of glucose variability were reported in 12 trials, most commonly standard deviation, and hypo- or hyperglycemia incidence^17^. Mean absolute glucose (MAG), mean amplitude of glycemic excursions (MAGE), glycemic lability index (GLI), M-value, J.Index, and continuous overlapping net glycemic action (CONGA) are different methods to calculate the GV^17,25,26^. These methods were designed to measure GV based on the magnitude of glycemic excursions, the time of exposure to glycemic excursions, or both. None of these methods have been adequately standardized; therefore, they are not widely used clinically, leading to a lack of evidence to support which method is most appropriate for use. We used continuous overlapping net glycemic action to describe indices of glycemic variability. The continuous overlapping net glycemic action (CONGA) is the standard deviation of the differences and measures the overall intra-day variation of glucose recordings. High CONGA values will therefore reflect increased glycemic excursions consistent with less stable control, and low CONGA values will reflect a stable glycemic control^26^. Further, compared to other methods CONGA does not ignore minor fluctuations in blood glucose values^27^.

The aim of our study is to assess the extent of glycemic variability (GV) for critically ill patients who received IIT therapy versus subcutaneous insulin (insulin sliding scale or ISS) and its impact on clinical outcomes.

## Methods

### Study design

**Figure 1:**
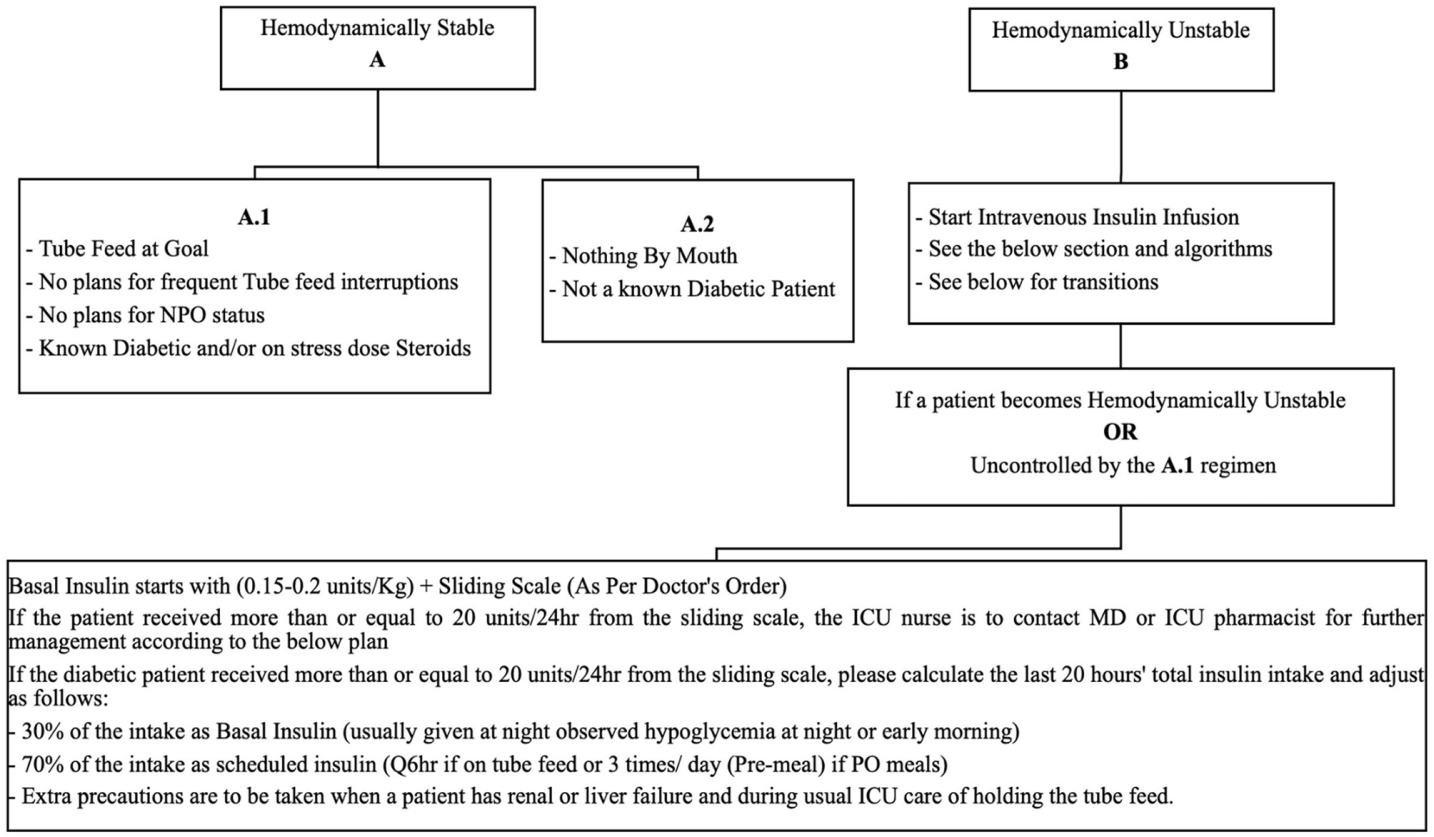
Insulin infusion therapy (IIT) protocol instructions.

A single-center, prospective cohort observational study involving all adult patients admitted to medical or surgical intensive care units at a tertiary academic center in Saudi Arabia from March 2021 to November 2021 and received insulin therapy for hyperglycemia management. Eligible patients were categorized into two sub-cohorts based on the regimen of insulin therapy (Insulin infusion therapy (IIT) or Insulin sliding scale (ISS)) (Fig. 1). The practitioner determined the decision to initiate the insulin infusion for patients who withhold food and fluids (NPO), those hemodynamically unstable or if blood glucose is not controlled with ISS as per local protocol. As per the protocol, the nursing staff has been directed to modify the insulin infusion rate based on the glucose value and the rate of glucose change. All patients were followed until they were discharged from the ICU or died during their ICU stay. The study was approved on January 31st, 2021, by King Abdulaziz University Hospital Ethics Committee—Institutional Review Board (IRB), Jeddah, Saudi Arabia, in January 2021 (Reference No: 18-21). All methods were performed in accordance with the guidelines and regulations issued by the National Committee of Bio and Med. Ethics—King Abdul Aziz City for Science and Technology. The study was conducted in accordance with the World Medical Association Declaration of Helsinki—Ethical Principles for Medical Research Involving Human Subjects (adopted 1964; updated 2013), national ethical regulations, and local institutional guidance of the study center. Due to the study's observational nature, informed consent from study participants was waived by King Abdulaziz University Hospital Ethics Committee—Institutional Review Board (IRB), Jeddah, Saudi Arabia.

### Study setting

This single-center study was conducted in mixed medical and surgical intensive care units (ICUs) at King Abdulaziz University Hospital (Jeddah, Saudi Arabia) which is considered an academic, tertiary care center that includes more than 800 beds. On average, a nurse takes care of one to two patients depending on the severity of the disease. The hyperglycemia and insulin therapy hospital protocol in adults ICU was implemented in January 2019 and set at a target glucose range of 140 to 180 mg/dL.

### Study participants

**Figure 2:**
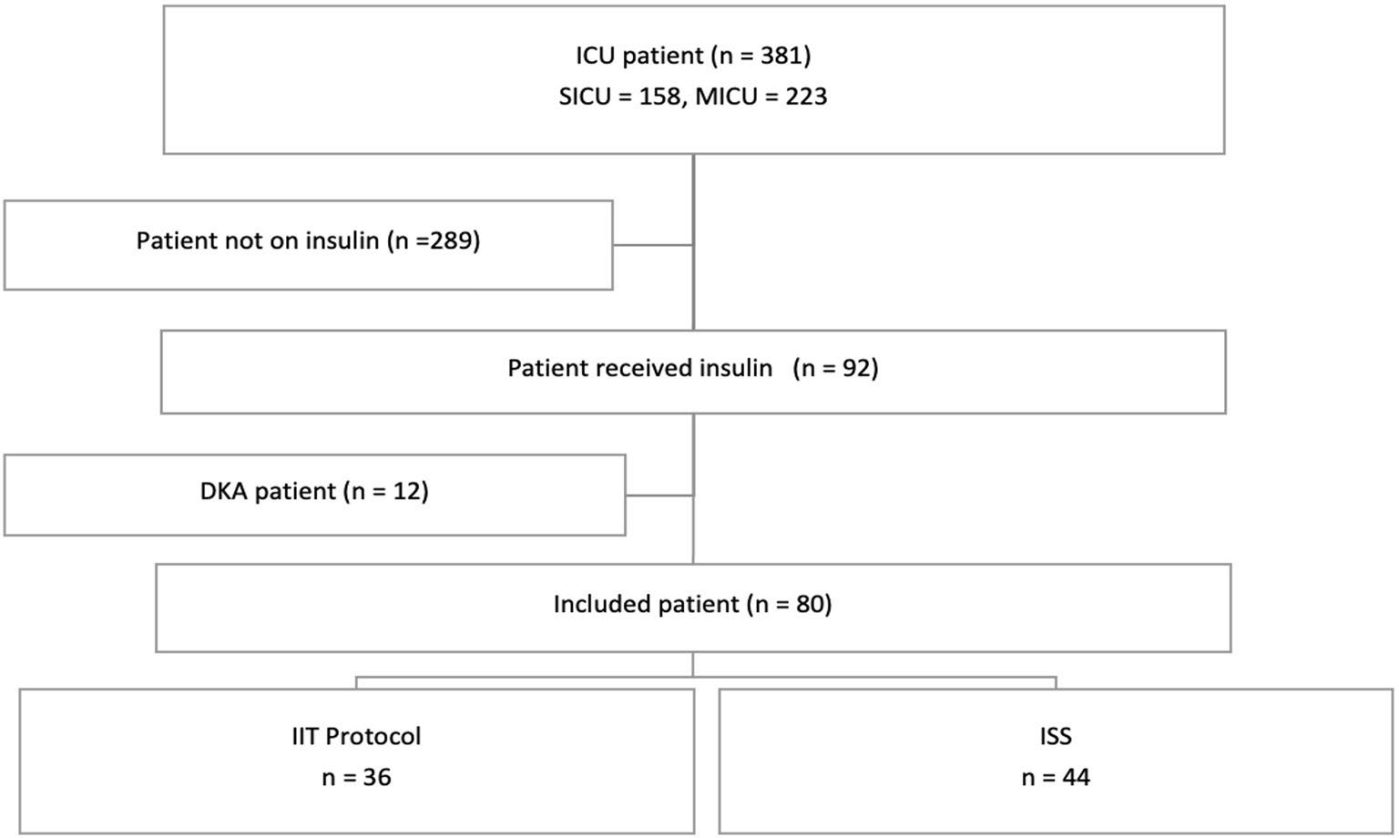
Flow chart of the patients included in the study.

All adult patients (18 years old or above) admitted to ICUs and received insulin therapy for hyperglycemia during the study period were assessed for eligibility. Patients were excluded if they were diagnosed with Diabetic Ketoacidosis (DKA), Hyperglycemic Hyperosmolar Syndrome (HHS), or if health records were unavailable (Fig. 2).

### Study outcomes

The primary outcome was to evaluate the extent of glycemic variability (GV) with the IIT protocol, compared with ISS using continuous overlapping net glycemic action (CONGA) and mean GV. While the secondary outcomes were the incidence of hypoglycemia, and the need for additional insulin doses outside the protocol (uncontrolled hyperglycemia refers to blood glucose not responding to scheduled insulin doses in ISS protocol or IIT protocol). Other outcomes such as ICU mortality, and ICU length of stay (LOS) were considered exploratory.

### Outcomes definition

#### Glycemic variability

Glucose variability (GV) refers to the extent of BG oscillations, in both magnitude and frequency including episodes of hypoglycemia and postprandial hyperglycemia^24^. The Global Variability (GV) was determined using the CONGA method, which was developed by Mcdonnell et al.^26^ in 2005. The CONGA is a metric that represents the standard deviation of the differences between glucose measurements taken at different times during the same day. It is used to quantify the overall intra-day variation of glucose recordings. High CONGA values will therefore reflect increased glycemic excursions consistent with less stable control, and low CONGA values will reflect a stable glycemic control^26^.

#### Hypoglycemia

Hypoglycemia is defined as BG less than or equal to 70 mg/dL (3.8 mmol/L), and severe hypoglycemia is defined as BG less than or equal to 40 mg/dL (2.2 mmol/L) and requires dextrose administration. Hypoglycemia events were assessed during the entire period of insulin administration.

### Data collection

We collected demographic information, ICU admission diagnosis, comorbidities, laboratory tests, severity scores (Acute Physiology and Chronic Health Evaluation II (APACHE II), Sequential Organ Failure Assessment (SOFA), and mechanical ventilation status. In addition, stress hyperglycemia, and regimen of insulin used were obtained by reviewing electronic and paper records.

Assessment of the blood glucose was conducted prospectively for the two groups. We include all patients who received insulin infusion at any time during their ICU stay. The times and levels of the blood glucose, doses of insulin, and route of insulin administration during ICU stay were identified via the Medication Administration Record. Besides, healthcare records were reviewed retrospectively to collect other information for all included patients. The blood glucose levels were monitored over a 48-hour period for patients who underwent ISS. In the IIT group, most patients received short-term insulin infusion, during which all blood glucose readings were recorded. The data was collected and reviewed by two evaluators, with any discrepancies resolved through consensus with a third investigator.

### Statistical analysis

Continuous variables were presented as mean with standard deviation (SD), while categorical variables as numbers with percentages. Student *t*-test was used for normally distributed continuous variables to study differences in means. For categorical variables, Chi-square and Fisher’s exact test were used to study differences in proportions.

Linear and logistic regression analyses were used to study group differences in glycemic indices and mortality, respectively adjusted for SOFA score, albumin level, age, gender, and BMI, that were hypothesized to have a potential clinical impact on GV and other outcomes. All model diagnostics were performed through an examination of residuals and multicollinearity to ensure adequate fit. In addition, the Hosemer–Lemeshow test was utilized to assess the logistic regression model. Logistic regression was preferred over other forms of survival analysis because of data constraints. A priori power analysis was conducted for linear regression to estimate sample size based on a small effect of 0.13 and alpha of 0.05. The results showed that a sample size of 81 was adequate to detect an association with a critical t equal to 1.17. We considered a p value of <0.05 statistically significant and used SPSS 29 for all statistical analyses.

### Ethics approval and informed consent

The study was approved on January 31st, 2021, by King Abdulaziz University Hospital Ethics Committee -Institutional Review Board (IRB), Jeddah, Saudi Arabia, in January 2021 (Reference No: 18-21). All methods were carried out in compliance with the guidelines and regulations issued by the National Committee of Bioethics and Medical Ethics at King Abdulaziz City for Science and Technology. Participants’ confidentiality was strictly observed throughout the study by using anonymous unique serial numbers for each subject and restricting data only to the investigators. Due to the study’s observational nature, informed consent from study participants was waived by King Abdulaziz University Hospital Ethics Committee—Institutional Review Board (IRB), Jeddah, Saudi Arabia.

## Results

### Demographic and clinical characteristics

During the nine-month study period, 381 critically ill patients were screened based on the eligibility criteria. Among them, only 80 patients were included (Fig. 2). Table 1 shows the demographics and baseline characteristics of the included patients. More than half of the patients in both groups were admitted to the medical intensive care unit. The most common reason for ICU admission was related to COVID-19 complications. Most of the patients who received ISS were male (61.4% vs. 41.7%), older (64.4 ± 14.3 years vs 59.7 ± 13.6 years), had a lower BMI (28.7 ± 4.5 vs 32.5 ± 13); however, were not statistically different between the two groups. Diabetes as a comorbidity and history of insulin use was higher in the IIT group compared with the ISS group but the difference was not statistically significant. As well as the APACHE II and SOFA scores within 24 h of ICU admission were significantly higher in patients who received the IIT protocol (*p*-value = 0.001, and *p*-value = 0.003, respectively).

**Table 1:** Baseline characteristics of the study sample (n = 80).

Characteristics	ISS (N = 44)	IIT Protocol (N = 36)	*p*-value
Age—years mean (SD)	64.36 (14.30)	59.67(13.60)	0.14
Female n (%)	17 (38.60)	21 (58.30)	0.08
BMI mean (SD)	28.67 (4.56)	32.47 (13)	0.10
Severity of illness			
APACHE II mean (SD)	18 (7.10)	24.4 (8.16)	0.001
SOFA mean (SD)	6 (3.22)	8.36 (3.65)	0.003
History of diabetes			
Previous diabetes n (%)	31 (70.50)	30 (83.30)	0.09
Previous insulin treatment n (%)	5 (11.14)	9 (25)	0.11
Admission location			
MICU n (%)	33 (75)	21 (58.30)	0.11
SICU n (%)	11 (25)	15 (41.70)	0.11
Reasons for ICU admission			
COVID-19 n (%)	21 (47.70)	15 (41.70)	0.59
Infectious n (%)	6 (13.60)	6 (16.70)	0.71
Cardiovascular n (%)	6 (13.60)	8 (22.20)	0.32
Neurological n (%)	5 (11.40)	3 (8.30)	0.65
Surgery n (%)	4 (9.10)	2 (5.60)	0.55
Other n (%)	2 (4.50)	2 (5.60)	0.84
Treatment during ICU stay			
Glucocorticoids n (%)	24 (54.50)	16 (44.40)	0.37
Beta-blocker n (%)	12 (27.30)	8 (22.20)	0.60
Vasopressor n (%)	11 (25)	12 (33.30)	0.41

### Glycemic variability (GV)

Table 2 shows the results of the association between GV indices and insulin approach (ISS vs IIT). Patients on the IIT protocol were found to be associated with a statistically significant mean decrease of 0.74 on CONGA values compared to patients on ISS usual care (0.74, 95% CI [0.28, 1.19], *p*-value = 0.002).

**Table 2:** Means and confidence intervals for differences between ISS and IIT protocol for GV mean and CONGA.

Dependent variable	Treatment group	Mean (SE)^a^	*p*-values (95% CI of the difference)
GV Mean	ISS	10.74 (0.28)	0.003 (− 2.25, − 0.49)*
	IIT protocol	12.43 (0.35)	
CONGA	ISS	2.87 (0.14)	0.002 (0.28, 1.19)*
	IIT protocol	2.13 (0.15)	

^a^Standard error.

### Hypoglycemia and uncontrolled hyperglycemia

There were no significant differences between the two groups regarding hypoglycemia that required correction, only one patient out of 36 patients who received insulin infusion required a bolus of 50% dextrose to correct severe hypoglycemia whereas three patients in the ISS group needed correction (6.8% vs 2.77%; *p*-value = 0.38). Moreover, about 27% of patients who received the ISS required additional insulin doses to manage their uncontrolled hyperglycemia. These additional doses were given as boluses subcutaneously and were added to the regularly scheduled doses on the insulin sliding scale.

### ICU-mortality and length of stay

In crude analysis, ICU Mortality was higher in the IIT group compared to the ISS group (55.6% vs 34.1%; *p*-value = 0.05). Moreover, at logistic regression analysis there was a higher odd of mortality among patients on IIT protocol by 90%; however, was not statistically significant (OR = 1.90, 95% CI [0.65, 5.53], *p*-value = 0.24) while adjusting for the covariates as shown in Table 3. On the other hand, the association between ICU length of stay (LOS) and insulin approach was not statistically significant between the two insulin approaches (0.72, 95% CI [− 5.98, 7.43], *p*-value = 0.83) as shown in Table 4.

**Table 3:** Logistic regression of Mortality.

Independent variable	Mortality	
	OR	*p*-value (95% CI Interval of OR)
Insulin approach (IIT)	1.90	0.24 (0.65, 5.53)
Covariates		
Gender (female)	1.28	0.63 (0.43, 3.19)
Age	0.98	0.28 (0.95. 1.02)
Albumin (g/L)	0.98	0.74 (0.93, 1.06)
SOFA Score	0.99	0.90 (0.87, 1.12)
BMI	1.00	0.97 (0.95, 1.05)

**Table 4:** Linear regression of glycemic variability indices and ICU length of stay.

Independent variable	Dependent variables					
	GV mean		CONGA		ICU length of stay	
	β^a^	*p*-value (95% CI for β)	β	*p*-value (95% CI for β)	β	*p*-value (95% CI for β)
Insulin approach (IIT)	1.27	0.014* (0.65, 2.57)	− 0.65	0.014* (− 1.16, − 0.14)	0.72	0.83 (− 5.98, 7.43)
Covariates						
Gender (female)	− 0.63	0.12 (− 0.54, 1.35)	− 0.23	0.35 (− 0.73, 0.26)	6.55	0.05* (0.01, 13.1)
Age	− 0.003	0.85 (− 0.048, 0.02)	0.01	0.12 (− 0.004, 0.04)	0.25	0.03* (0.03, 0.47)
Albumin	− 0.01	0.73 (− 0.11, 0.01)	− 0.01	0.42 (− 0.05, 0.02)	0.27	0.21 (− 0.16, 0.69)
SOFA Score	0.12	0.09 (− 0.07, 0.21)	0.03	0.40 (− 0.04, 0.11)	0.01	0.98 (− 0.95, 0.98)
BMI	− 0.01	0.80 (− 0.05, 0.04)	− 0.003	0.79 (− 0.03, 0.02)	0.26	0.11 (− 0.06, 0.58)

^a^Beta coefficient.

## Discussion

In this study, we compared the effect of different approaches to insulin administration (IIT and ISS) on blood glucose variability in critically ill patients. We observed a significant difference in glycemic variability (GV) between the two groups. We found that the use of IIT for stress hyperglycemia was associated with lower GV than ISS in critically ill patients. No significant difference was noted in both mortality and ICU length of stay between the groups. Moreover, the ISS group has a higher frequency of hypoglycemia required correction compared to the IIT group, but this difference did not reach statistical significance.

There is no established “gold standard” method to evaluate GV. Previous investigators have suggested the use of different ways to measure glycemic variation including the mean and standard deviation of the blood glucose, continuous overlapping net glycemic action (CONGA)^20^, J.Index^25^, and mean amplitude of glycemic excursions (MAGE)^28^. In our study, we targeted CONGA values to describe indices of GV, since it does not ignore minor fluctuations in blood glucose values^27^. Additionally, other methods such as the mean of daily differences (MODD) is calculated based on the mean of the absolute differences between glucose values at the same time on two consecutive days. Since our patients have received IIT for a short time, less than 48 hours, utilizing parameter such as MODD would not be appropriate in this scenario, and we opted to utilize CONGA as it is a better fit to our patients’ settings. On the other hand, additional methods such as high blood glucose index (HBGI), and low blood glucose index (LBGI) are limited as they are only a predication for severe hypoglycemia or severe hyperglycemia, and both do not reflect the Glucose variability. The results were consistent, the GV was significantly higher in the ISS group. Patients on the IIT protocol were found to be associated with a statistically significant mean decrease of 0.65 on CONGA values compared to patients on ISS usual care (− 0.65, 95% CI [− 1.16, − 0.14], *p*-value = 0.014, adj. R^2^ = 0.09). This result was consistent with Clergeau’s study in critical care patients which also confirmed the significant reduction of GV with insulin infusion by using the mean amplitude of glycemic excursions (MAGE)^28^.

The cumulative number of comorbidities can influence the overall health status of the patient population including their response to stress as well as developing stress hyperglycemia which may require insulin therapy and having these patients more prone for glycemic variability, however both groups were comparable in terms of comorbidities at baseline. Within our study population, COVID-19 exhibits a substantial prevalence across the groups, with rates of 42.7% observed in the IIT group and 47.7% in the ISS group. COVID-19 infection can increase the secretion of stress hormones such as glucocorticoids and catecholamines that may increase blood glucose^29^. The study of Montefusco et al. found that COVID-19 is associated with higher glycemic variability, as shown by a higher coefficient of variability and higher standard deviation in ICU patients^30^. A recent retrospective study found that patients with COVID-19 had significantly higher mean daily glucose values and greater mean daily glucose variability. However, there was no significant difference in the proportion of patients with an episode of hypoglycemia (< 70 mg/dL) during the ICU stay^31^.

Shock status, edema, and vasopressors may interfere with cutaneous blood flow resulting in decreased bioavailability in critically ill patients^32^. This may cause insulin accumulation, increased insulin doses, and a risk of hypoglycemia simultaneously. In our study, patients who received the IIT protocol had a lower incidence of hypoglycemia that required correction. The correction was necessary if the blood glucose level fell below 70 mg/dL (3.9 mmol/L) after stopping the insulin infusion or holding the dose of the insulin sliding scale. Giving 12.5 g of intravenous (IV) bolus of 50% dextrose. However, if the blood glucose level is below 40 mg/dL (2.2 mmol/L), 25 g of intravenous 50% dextrose should be given. Blood glucose levels must be monitored 15 min and 30 min after an episode of hypoglycemia.

Only three patients in the IIT group needed to change the algorithm of insulin therapy to the higher insulin doses algorithm. While twelve patients in the ISS group needed additional doses of insulin. This is consistent with Tran et al.^24^ study that the insulin infusion was observed to have a lower occurrence of hypoglycemia compared to subcutaneous insulin (p < 0.01). Conversely, Khan et al.^33^ a quasi-experimental study demonstrated that hypoglycemia is more common in patients who received intravenous insulin infusion than in the subcutaneous group. Nonetheless, Bhurayanontachai et al.^8^ showed that there is no association between severe hypoglycemia events in critically ill patients and the route of insulin administration, whether intravenous or subcutaneous.

Our findings showed that both ICU length of stay and mortality were similar between the groups with an estimated mean difference of 2.72 days (beta coefficient (95% CI); 0.72, *p*-value = 0.83). On the contrary, Kim et al.^34^ a retrospective single-center study conducted in the intensive care unit to assess the effect of GV on 28-day mortality and ICU length of stay demonstrated that early GV is associated with higher mortality and a longer ICU length of stay. Additionally, studies conducted by Bruginski et al.^35^ and Mendez et al.^36^ in non-critically ill patients confirmed GV independently increased the duration of hospital length of stay. In contrast, Aldorzi et al.^16^ did not find any significant correlation between glycemic fluctuations and ICU or hospital length of stay *p*-value (0.58 vs 0.36) respectively in a medical-surgical intensive care unit. Our study was not specifically designed to assess the effects of GV reduction on ICU length of stay and mortality, with a relatively small sample size which might limit drawing a robust conclusion. However, an a priori power analysis was conducted for linear regression to estimate sample size based on a small effect of 0.13 and alpha of 0.05. The results showed that a sample size of 81 was adequate to detect an association with a critical t equal to 1.17.

Secondly, we depend on venous blood samples assay to determine blood glucose, which may underestimate serum glucose concentration. Third, we retrospectively collect blood glucose levels and insulin doses from medical records. We couldn’t rule out the possibility of bias as we compared the severity of illness at baseline, not during the ICU stay. Fourth, we acknowledge that CONGA value is not the gold standard to assess GV in the ICU setting. However, to our knowledge, the CONGA is the best available method to evaluate intra-day glycemic variability.

## Conclusion

Our study demonstrated that the IIT is associated with a significant decrease in GV in critically ill patients. There were no significant differences in other outcomes such as hypoglycemia and uncontrolled hyperglycemia. Additional studies with a larger sample size are needed to confirm our findings and elaborate on IIT's based protocol on important ICU outcomes such as mortality and ICU length of stay.

## Data Availability

The datasets supporting the conclusions of this article are available from the corresponding author on reasonable request.
